# Effects of an urban cable car intervention on physical activity: the TrUST natural experiment in Bogotá, Colombia

**DOI:** 10.1016/S2214-109X(23)00274-7

**Published:** 2023-07-18

**Authors:** Laura Baldovino-Chiquillo, Olga L Sarmiento, Gary O’Donovan, Maria A Wilches-Mogollon, Andres F Aguilar, Alberto Florez-Pregonero, Paola A Martínez, Julian Arellana, Luis A Guzmán, Goro Yamada, Daniel A Rodriguez, Ana V Diez-Roux

**Affiliations:** a School of Medicine, Universidad de los Andes, Bogotá, Colombia; b BrainLat, Universidad Adolfo Ibáñez, Santiago, Chile; c Instituto Masira, Universidad de Santander, Bucaramanga, Colombia; d Department of Industrial Engineering, School of Engineering, Universidad de los Andes, Bogotá, Colombia; e Grupo de Sostenibilidad Urbana y Regional, Department of Civil and Environmental Engineering, School of Engineering, Universidad de los Andes, Bogotá, Colombia; f School of Education, Pontificia Universidad Javeriana, Bogotá, Colombia; g Department of Civil and Environmental Engineering, Universidad del Norte, Barranquilla, Colombia; h Urban Health Collaborative, Dornsife School of Public Health, Drexel University, Philadelphia, PA, USA; i Department of Epidemiology and Biostatistics, Dornsife School of Public Health, Drexel University, Philadelphia, PA, USA; j Department of City and Regional Planning and Institute for Transportation Studies, University of California Berkeley, Berkeley, CA, USA

## Abstract

**Background:**

Cable cars are part of the transport system in several cities in Latin America, but no evaluations of their effects on physical activity are available. TransMiCable is the first cable car in Bogotá, Colombia, and the wider intervention includes renovated parks and playgrounds. We assessed the effects of TransMiCable and the wider intervention on physical activity.

**Methods:**

The Urban Transformations and Health natural experiment was a prospective quasi-experimental study conducted from Feb 1, 2018, to Dec 18, 2018 (baseline, pre-intervention) and from July 2, 2019, to March 15, 2020 (post-intervention follow-up) in the TransMiCable intervention area (Ciudad Bolívar settlement) and a control area without TransMiCable (San Cristóbal settlement). A multistage strategy was used to sample households in each area, with one adult (aged ≥18 years) per household invited to participate. Eligible participants had lived in the intervention or control areas for at least 2 years and were not planning to move within the next 2 years. Physical activity was assessed among participants in the intervention and control areas before and after the inauguration of TransMiCable in Ciudad Bolívar with the International Physical Activity Questionnaire (long form) and with wearable accelerometers. Complete cases (those with baseline and follow-up data) were included in analyses. Respondents were classed as being physically active if they met 2020 WHO guidelines (≥150 min per week of moderate activity, ≥75 min per week of vigorous activity, or equivalent combinations); and accelerometery data were classified with the Freedson cut-points for adults. Data were also gathered in zonal parks (area ≥10 000 m^2^) and neighbourhood parks (area <10 000 m^2^) in the intervention and control areas by direct observation with the System for Observing Play and Recreation in Communities, to assess levels of physical activity before and after the TransMiCable intervention. Multilevel regression models were used to assess changes in physical activity associated with the TransMiCable intervention.

**Findings:**

Physical activity questionnaires were completed by 2052 adult participants (1289 [62·8%] women and 763 [37·2%] men; mean age 43·5 years [SD 17·7]) before the inauguration of TransMiCable. After the inauguration, the follow-up (final) questionnaire sample comprised 825 adults in the intervention group and 854 in the control group, including 357 adults in the intervention group and 334 in the control group with valid accelerometery data. 334 (40·5%) of 825 participants in the intervention group reported levels of physical activity that met the 2020 WHO guidelines during walking for transport before the intervention, and 426 (51·6%) afterwards (change 11·1 percentage points [95% CI 6·4 to 15·9]). A similar change was observed in the control group (change 8·0 percentage points [3·4 to 12·5]; adjusted odds ratio [OR] for the time-by-group interaction, intervention *vs* control group: 1·1 [95% CI 0·8 to 1·5], p=0·38). Time spent doing moderate-to-vigorous physical activity, measured with accelerometers, did not change in the intervention group after the inauguration of TransMiCable (change –0·8 min per day [–4·6 to 3·0]) and did not change compared with the control group (adjusted β for the time-by-group interaction: 1·4 min per day [95% CI –2·0 to 4·9], p=0·41). Moderate-to-vigorous physical activity was 52·1 min per day (SD 24·7) before and 59·4 min per day (35·2) after the inauguration of TransMiCable in new regular users who reported using TransMiCable during mandatory trips for work or education (n=32; change 7·3 min per day [–22·5 to 7·9]). After the intervention, an increase in the proportion of male individuals engaging in moderate or vigorous physical activity was observed in a renovated zonal park (adjusted OR for the time-by-group interaction, intervention *vs* control park: 2·7 [1·1 to 6·8], p=0·033). Female users of a renovated neighbourhood park were less likely to become engaged in moderate or vigorous physical activity than female users of the control area neighbourhood park (adjusted OR for the time-by-group interaction: 0·4 [0·1 to 0·6], p=0·019).

**Interpretation:**

It is encouraging that walking for transport remained high in the TransMiCable intervention area when the use of private motorised transport had increased elsewhere in Bogotá. In low-income urban areas, where transport-related walking is a necessity, transport interventions should be focused on efforts to maintain participation in active travel while improving conditions under which it occurs.

**Funding:**

Wellcome Trust (as part of the Urban Health in Latin America project); Bogotá Urban Planning Department; Ministry of Science, Technology, and Innovation of Colombia; Universidad de Los Andes; Fundación Santa Fe de Bogotá; and Universidad del Norte.

**Translation:**

For the Spanish translation of the abstract see Supplementary Materials section.

## Introduction

Physical activity is a major protective factor for morbidity and mortality.^1^ The promotion of physical activity might be beneficial to meeting 15 of the 17 UN Sustainable Development Goals.^2^ However, one in four adults is physically inactive globally, with considerable variability across countries.^3^ In Latin America, an estimated 39% of the adult population were insufficiently active between 2001 and 2016.^3^ Public transport interventions have been associated with increased transport-related physical activity in studies conducted in cities mainly in Europe and North America.^4^, ^5^ However, evidence on the effects of such interventions in Latin America is scarce.

Latin America is one of the most urbanised and unequal regions in the world, with large populations living in low-income settlements.^6^ These populations often face disadvantages, including poverty, violence, few recreational spaces, and poor access to transport.^7^ Therefore, for these populations, the decision to use active or multimodal transport is a necessity rather than a choice.^8^ Latin America is also an innovative region in mobility policies such as those for cable cars.^9^ At least 13 cities in Latin America have implemented cable cars as part of their public transport systems to overcome accessibility and connectivity issues affecting residential areas located on steep slopes, most of which are low-income settlements.^9^ However, to our knowledge, the effects of cable cars on physical activity have not been investigated to date.

One of the most recent cable cars to have been implemented in Latin America is TransMiCable in Colombia. This cable car was introduced in 2018, and connects Ciudad Bolívar, a low-income settlement located on steep hillsides in Bogotá, with the city's bus rapid transit system.^10^, ^11^ TransMiCable was conceived because travelling by directly traversing the hillside was regarded as a more viable solution than rehousing people and building roads. The cable car was used by around 7·5 million people in 2019^12^ and is part of a comprehensive urban transformation project.^10^, ^11^

**Research in context**

**Evidence before this study**

To identify previous studies examining the association between the implementation of cable cars or new public transport systems and physical activity, we searched PubMed and SciELO for articles published from database inception up to Nov 15, 2020, using the following search terms without language restrictions: (“cable car” OR “ropeway” OR “transportation” OR “public transit”) AND (“physical activity”) AND (“impact” OR “effect” OR “systematic review” OR “meta-analysis”). Although we found two recent meta-analyses evaluating the association between new transport interventions and physical activity, none of the included studies evaluated interventions of cable car systems. Interventions in the previous studies included light rail transit systems and bus rapid transit lines implemented in middle-income and high-income areas. According to these meta-analyses, new public transport interventions were associated with an increase in light-to-moderate physical activity levels of about 30 min per week, an increase in transport-related physical activity of about 7 min per week, and a decrease in total physical activity of about 37 min per week.

**Added value of this study**

To the best of our knowledge, this study is the first to assess the effects of a cable car intervention on physical activity. We found that the physical activity of adult residents of the TransMiCable cable car intervention area was maintained to a high level after the inauguration of TransMiCable, and was similar to the activity level of residents in the control area, on the basis of self-reported physical activity and accelerometery. In the intervention area, 40·5% of participants reported physical activity levels that met guideline-recommended amounts (2020 WHO guidelines) before the inauguration of TransMiCable, and 51·6% after the inauguration, from transport-related walking alone, with no difference between the intervention and control groups. Observed physical activity increased among users of one of the renovated parks that was part of the wider intervention of TransMiCable, but only among males.

**Implications of all the available evidence**

Most research on transport interventions and physical activity has been done in high-income areas. The findings of our study highlight the importance of integrated urban transport interventions in efforts to maintain physical activity levels in low-income areas in the Global South. In these areas, where physical activity is a necessity, transport policies should be accompanied by efforts to maintain participation in active travel by improving the conditions under which this travel occurs.

The 2022 *Lancet Global Health* Series on urban design, transport, and health emphasised that governments risked committing to unhealthy and unsustainable urban systems if policies and interventions were not properly evaluated.^13^ To transition towards healthy and sustainable cities in low-income and middle-income countries, an increased capacity in research is needed to fill the gap in research about urban design and transport, and links to health.^14^ The TransMiCable evaluation study, Urban Transformations and Health (TrUST, according to its initials in Spanish),^12^ conducted by the Urban Health in Latin America project (known as SALURBAL), offers a unique opportunity to investigate the effects of an integrated cable car system on health, social, and environmental outcomes in a low-income area in Latin America. The TrUST natural experiment was designed in conjunction with community leaders, the mayor's office of Bogotá, and with other stakeholders.^12^ The aim of TrUST was to assess the effects of the TransMiCable project on physical activity. A hypothesis of the study was that physical activity would increase with TransMiCable, particularly walking for transport, because the cable car is faster, safer, and more comfortable than existing modes of transport. A further hypothesis was that physical activity would increase among users of the renovated parks that were part of the wider TransMiCable intervention.^12^

## Methods

### Study setting

TrUST was conducted in Bogotá, Colombia. The intervention area was the settlement of Ciudad Bolívar, which is the area that TransMiCable serves. The control area was the settlement of San Cristóbal, where implementation of TransMiCable is planned in 2025. The intervention and control areas were deemed appropriate because they have similar topographies, they are generally of low socioeconomic status (per the national socioeconomic stratification of Colombia, most of the populations are in socioeconomic stratum 1 or 2)^15^, they have high crime levels,^16^ and there are geographical barriers between them that limit population crossover. Notably, the intervention and control areas are separated by 4·8 km of barren and mountainous land, and they have independent transport routes and bus stations (appendix 2 p 3).^12^

**Figure 1:**
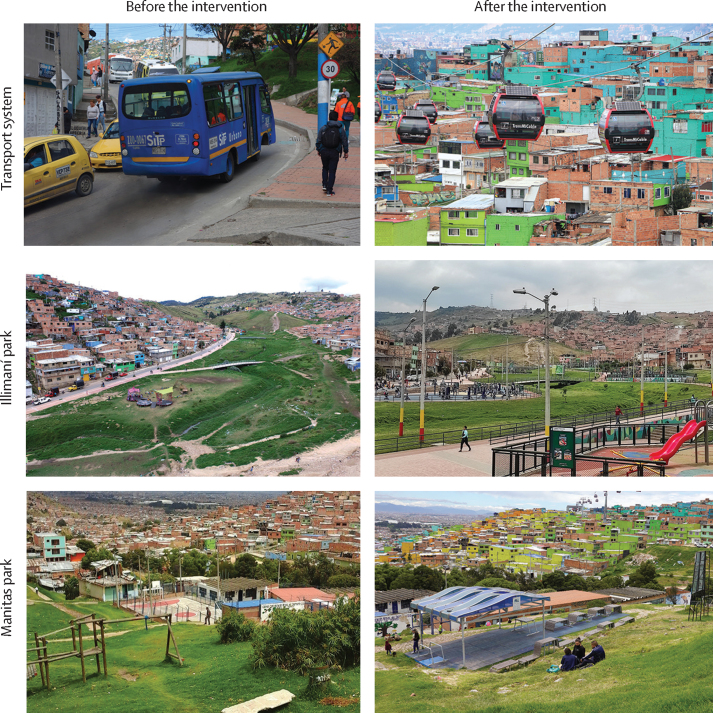
Intervention neighbourhoods before and after inauguration of the TransMiCable cable car project The wider intervention of the TransMiCable project includes the renovation of public parks. The zonal park assessed in this study (Illimaní park) was enlarged and renovated with outdoor gyms, playgrounds, and sports pitches. The neighbourhood park assessed in this study (Manitas park) was renovated with tables and seating for social gatherings. Additional photographs and a video of the TransMiCable intervention are available in appendix 2 (p 3).

TransMiCable was implemented in Dec 27, 2018, in Ciudad Bolívar.^12^ The cable car has four stations and 163 cabins. TransMiCable was the main component of an urban intervention that included a library, a tourism office, a local history museum, a citizen service office, three local markets, three community centres, and renovated public parks (figure 1; appendix 2 p 4).^12^ The intervention is ongoing and two bike trails and two sport and recreation centres are planned.

### Study design and participants

TrUST used a prospective, quasi-experimental design with a mixed-methods approach described in detail previously.^12^ In this manuscript, we focused on the reporting of quantitative results for the effects on physical activity outcomes. The intervention group in Ciudad Bolívar included households within an 800-m radius buffer of each TransMiCable station. The control group in San Cristóbal included households within an 800-m buffer of the planned locations for the future cable car stations (appendix 2 p 5). The study was approved by the ethics committee of the Universidad de Los Andes (record numbers 806-2017, 977-2019, and 994-2019). All participants provided written informed consent.

In each household, one adult aged 18 years or older was invited to participate. Eligible participants had to have lived in the intervention or control areas for at least 2 years and were not planning to move within the next 2 years. A multistage sampling strategy was used.^12^ First, within neighbourhoods in each 800-m buffer, blocks were selected with a probability proportional to the density of households. Second, every third household was systematically selected. Third, we randomly selected one adult per household with use of a random number table. Everyone who participated in the study was given an incentive (a tote bag and a US$3 gift card) and was included in a draw for a $150 gift card.^12^

In the intervention area, a zonal park (Illimaní park) and a neighbourhood park (Manitas park) that were renovated as part of the wider intervention were also included (figure 1). Zonal parks are those with an area of at least 10 000 m^2^. Neighbourhood parks are those with a smaller area to a minimum of 1000 m^2^. In the control area, a zonal park (Moralba park) and a neighbourhood park (La Victoria park) were selected. The zonal parks and neighbourhood parks in both areas were similar distances from the TransMiCable stations (or planned stations in the control area; appendix 2 p 5).^12^ The parks were similar in terms of the type of areas for physical activity (appendix 2 p 10).

### Procedures

Baseline data in adults were collected before the inauguration of TransMiCable from Feb 1, 2018, to Dec 18, 2018. Follow-up data were collected after the inauguration from July 2, 2019, to March 15, 2020 (appendix 2 p 5). Physical activity in individuals was assessed with questionnaires and accelerometers. The long form of the International Physical Activity Questionnaire was used for all participants to assess physical activity in transport (ie, transport-related walking) and leisure-time domains.^17^ Participants were classified as physically active if they met 2020 WHO guidelines of at least 150 min per week of moderate activity, at least 75 min per week of vigorous activity, or equivalent combinations.^18^ Wearable accelerometer devices (models wGT3X-BT, GT3X, or GT3X+; ActiGraph, Pensacola, FL, USA) were used in a subsample of individuals to objectively assess minutes per day of moderate-to-vigorous physical activity, light physical activity, and sedentary time. Participants were asked to position the accelerometer over the right midaxillary waistline and to wear it during waking hours for at least 7 consecutive days. Accelerometer counts were aggregated into 60-s epochs. An algorithm programmed in R (version 3.3.2) was developed for wear-time validation. A minimum of 3 weekday days and 1 weekend day with at least 8 h of wear-time each day were required for validity. Data were scored with the Freedson accelerometer cut-points for adults (sedentary time, 0–99 counts per minute [CPM]; light physical activity, 100–1951 CPM; moderate activity, 1952–5724 CPM; and vigorous activity, ≥5725 CPM; with moderate and vigorous physical activity grouped in a single category).^19^

Sociodemographic characteristics, transport mode, and travel time were assessed with a questionnaire, as detailed previously.^12^ The road network distance between the participant's household and the nearest bus rapid transit station was estimated with a shortest path algorithm, according to the household and station geolocations, with use of ArcGIS software (version 10.3). Built environment characteristics were assessed with secondary local data from the Spatial Data Infrastructure for Bogotá, including the terrain slope, road intersection density, and park density. For this purpose, a 500-m road network buffer was created around the georeferenced household. The slope of the terrain was computed with the triangulated irregular network model, which represents terrain surfaces irregularly distributed to accommodate areas of high variability in the surface every 5 m. Road intersection density was calculated as the number of intersections per km^2^. Park density was calculated as the area in m^2^ of parks within each household's 500-m buffer area.

Data collection in parks was also done during the baseline and follow-up periods. Physical activity in parks was assessed with the System for Observing Play and Recreation in Communities.^20^ This tool has been used in Latin America and has been shown to be reliable.^21^ Parks were divided into target areas according to the physical activity available (eg, playgrounds, outdoor gyms, and football fields). In the intervention area, the zonal park had 22 target areas and the neighbourhood park had four target areas. In the control group, the zonal park had 15 target areas and the neighbourhood park had eight target areas. Two trained observers visited each park's target areas for all 7 days of the week (not consecutively) in the morning (0900–1200 h) and the afternoon (1300–1600 h). Details on the training of observers are provided in appendix 2 (p 6). Data were collected during 1372 observation visits before the cable car intervention (n=686 visits) and after the intervention (n=686 visits). Data collected included the number of park users according to sex, age group, and physical activity levels. The assigned age groups were children (age 0–12 years), adolescents (age 13–20 years), adults (age 21–59 years), or older adults (age ≥60 years) according to observer judgement. Physical activity level was defined as sedentary (lying down, sitting, or standing), moderate (eg, walking slowly), or vigorous (eg, walking fast, running, aerobic classes, or playing football). Observers also noted whether the target areas of the park were accessible, usable, equipped, supervised, organised, or empty.^20^ The quality of equipment and amenities was assessed with the Physical Activity Resource Assessment instrument,^22^ which qualifies the conditions of parks into six domains (features for physical activity practices; amenities; incivilities; services; accessibility; and safety; appendix 2 p 6).

### Statistical analysis

The target sample size of individuals in the intervention and control groups was estimated with a two-tailed test for mean difference to detect changes equivalent to standardised mean differences in self-reported minutes per day of moderate-to-vigorous physical activity from 0·3 to 0·4 (physical activity questionnaire sample) and in objective minutes of moderate-to-vigorous physical activity from 0·2 to 0·4 (accelerometery sample), on the basis of previous literature.^23^, ^24^, ^25^ To achieve a power of at least 80%, the target sample size for the questionnaire sample was estimated at 800 adults per group, considering a response rate of 70%, and for the accelerometery sample was estimated at 426 adults per group, considering a response rate of 80%.

We calculated descriptive statistics for covariates and physical activity outcomes. Outcomes of interest were the proportions of individuals who self-reported levels of physical activity that met 2020 WHO guidelines during transport-related walking and leisure time; minutes per day of moderate-to-vigorous physical activity, light physical activity, and sedentary time measured by accelerometery; and observed physical activity levels among park users measured by direct observation. We compared the changes in physical activity outcomes before and after the TransMiCable inauguration in the intervention and control groups using the paired *t*-test for continuous variables and the χ^2^ test for categorical variables. Changes were also assessed in a subgroup of new regular users of the TransMiCable, defined as individuals who reported using TransMiCable on at least one part of their mandatory trips for work or education. To assess the effect of TransMiCable on individual physical activity, we fitted multilevel regression models (logistic model for the outcomes of meeting physical activity guidelines based on the questionnaire; and linear model for the outcomes of minutes per day of physical activity based on accelerometery) with random intercepts for individuals. To assess the effect of the TransMiCable project on park-level physical activity, we fitted multilevel logistic regression models with park users’ data and random intercepts for the target area and period of the day. All models included the main effects of time (before and after the TransMiCable inauguration) and study group (intervention or control), as well as a time-by-group interaction. The time-by-group interaction term was used to assess the effect of TransMiCable by comparing the changes in outcomes over time between the intervention and control groups. The interaction effects are reported as β (95% CI) for multilevel linear regression models, and as odds ratio (OR; 95% CI) for multilevel logistic regression models. Individual-level models were adjusted for sociodemographic characteristics (age, sex, occupation, marital status, and education), distance to a bus rapid transit station, and the slope of the terrain (unadjusted models are reported in appendix 2 pp 8–9). Complete cases (those with baseline and follow-up data) were included in the analyses. We also conducted sensitivity analyses on the individual-level linear models with log-transformed outcomes due to the non-normality of accelerometery data, and a stratified analysis by sex. The park-level models were adjusted for the day of the week (weekday or weekend day) and the analysis was stratified by sex. All observed data were used for the park-level regression models. Model equations are described in appendix 2 (pp 6–7). Analyses were conducted with Stata (version 17) and R (version 3.3.2).

### Role of the funding source

The funders of the study had no role in study design, data collection, data analysis, data interpretation, or writing of the report.

## Results

**Figure 2:**
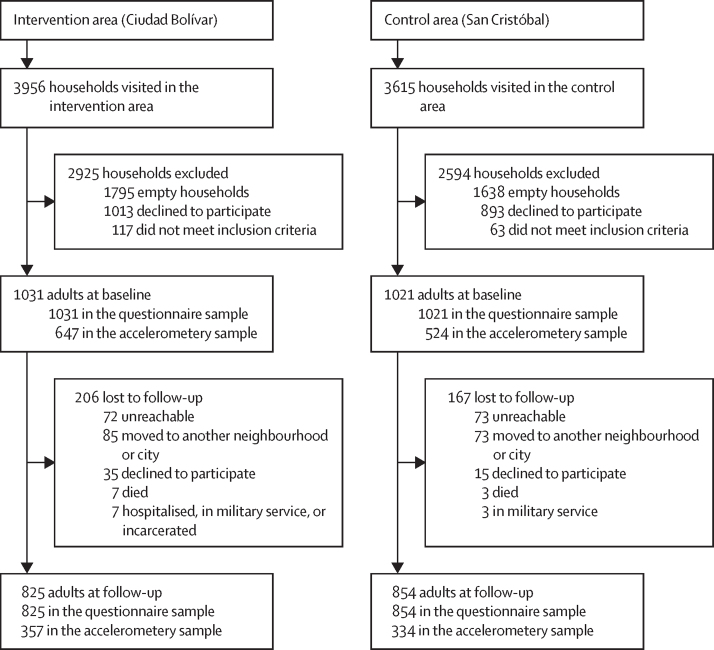
Sampling of households and participants In each household, we invited one adult aged 18 years or older to participate in the study. Eligible participants had lived in the intervention or control area for at least 2 years and were not planning to move within the next 2 years.

After sampling of households, physical activity questionnaires were completed by 2052 adult participants (1031 in the intervention area and 1021 in the control area) at baseline, before the inauguration of TransMiCable (figure 2). Accelerometery data were collected from 647 (62·8%) of the 1031 participants in the intervention area and 524 (51·3%) of the 1021 participants in the control area before the TransMiCable inauguration. After the inauguration, 825 (80·0%) participants in the intervention area and 854 (83·6%) in the control area completed follow-up physical activity questionnaires. Of participants with baseline accelerometery data, 357 (55·1%) of 647 in the intervention area and 334 (51·6%) of 524 in the control area provided valid follow-up accelerometery data. The individuals who completed baseline and follow-up questionnaires comprised the complete case sample for questionnaire-based outcomes, and those who provided valid baseline and follow-up accelerometery data comprised the complete case sample for accelerometery-based outcomes.

The characteristics of participants, transport choices, and features of the built environment before the inauguration of TransMiCable were summarised (table). Of the 2052 participants, 1289 (62·8%) were women and 763 (37·2%) were men, and mean age was 43·5 years (SD 17·7). 1061 (51·7%) participants were married or living with a partner. Most participants attained a maximum of elementary (primary level) or high-school (secondary level) education and reported a monthly household income of two or fewer minimum wage salaries. 1137 (55·4%) participants were working or studying. Compared with the control group, the intervention group had a larger proportion of individuals with a lower education level and monthly salary, and a smaller proportion of individuals who were working or studying. The main transport mode for mandatory trips (defined as travel for work or education) was public transportation. Self-reported mean travel time during mandatory trips (one way) was 110·0 min (SD 67·3) in the intervention group and 89·9 min (54·1) in the control group. After the inauguration of TransMiCable, 95 (11·9%) of 797 participants in the intervention group who undertook mandatory trips reported using TransMiCable regularly, 617 (74·8%) of 825 reported using the cable car at least once, and the mean travel time for mandatory trips decreased to 90·2 min (SD 53·9; n=398) in the intervention area. Hilly terrain, high road intersection density, and low park density characterised the built environment of the intervention and control areas (table).

**Table:** Participant characteristics, travel time, and mode of transport before the inauguration of TransMiCable, and built environment of the study areas

		Intervention (n=1031)*	Control (n=1021)*
**Sociodemographic characteristics**			
Age, years		1031; 44·3 (18·1)	1021; 42·8 (17·3)
Sex			
	Female	668 (64·8%)	621 (60·8%)
	Male	363 (35·2%)	400 (39·2%)
Civil status			
	Single	214 (20·8%)	331 (32·4%)
	Married or with partner	551 (53·4%)	510 (50·0%)
	Divorced, separated, or widowed	266 (25·8%)	180 (17·6%)
Education level†			
	Elementary or lower	399/1030 (38·7%)	256 (25·1%)
	High school	466/1030 (45·2%)	514 (50·3%)
	Technical, college, or graduate	165/1030 (16·0%)	251 (24·6%)
Monthly household income‡			
	≤1 minimal wage (≤USD$264)	556/1023 (54·3%)	350/984 (35·6%)
	>1 to ≤2 minimal wages (US$265–528)	386/1023 (37·7%)	485/984 (49·3%)
	>2 minimal wages (≥US$529)	81/1023 (7·9%)	149/984 (15·1%)
Occupation			
	Working	496/1029 (48·2%)	528/1019 (51·8%)
	Studying	42/1029 (4·1%)	71/1019 (7·0%)
	Household activities	322/1029 (31·3%)	294/1019 (28·8%)
	Not working	169/1029 (16·4%)	126/1019 (12·4%)
**Transportation**			
Main transport mode for mandatory trips§			
	Public	493/803 (61·4%)	554/823 (67·3%)
	Public and active	174/803 (21·7%)	168/823 (20·4%)
	Public and private	34/803 (4·2%)	21/823 (2·5%)
	Public and informal	52/803 (6·5%)	12/823 (1·5%)
	Private	50/803 (6·2%)	68/823 (8·3%)
Travel time for mandatory trips,§ min		375; 110·0 (67·3)	396; 89·9 (54·1)
**Built environment**			
Slope of the terrain, %		NA; 9·9 (3·6)	NA; 6·4 (1·8)
Road intersection density, n/km^2^		NA; 578·2 (128·7)	NA; 415·6 (121·6)
Park density, m^2^/m^2^¶		NA; 0·06 (0·04)	NA; 0·05 (0·03)

Data are n; mean (SD) or n (%). NA=not applicable.

* Participant numbers might not sum to the total group size due to missing data; denominators are shown when participant data were missing.

† Education levels in Colombia equivalent to primary level education (elementary), secondary level education (high school), and higher education (technical, college, or graduate).

‡ Minimum wage as defined by the Colombian Ministry of Labour^26^ with use of a mean exchange rate for 2018 of USD$1=2956·36 Colombian pesos.

§ Mandatory trips defined as travel for work or education (one way). Public transport refers to state-owned modes of transport, such as local buses and the bus rapid transit system. Active transport refers to walking or cycling. Private transport refers to privately owned modes of transport, such as privately owned cars and motorbikes, or taxis. Informal transport refers to unofficial modes of transport that supplement public transport routes, such as unofficial local buses.

¶ The area of parks (m^2^) within each household's 500-m road network buffer area (m^2^).

**Figure 3:**
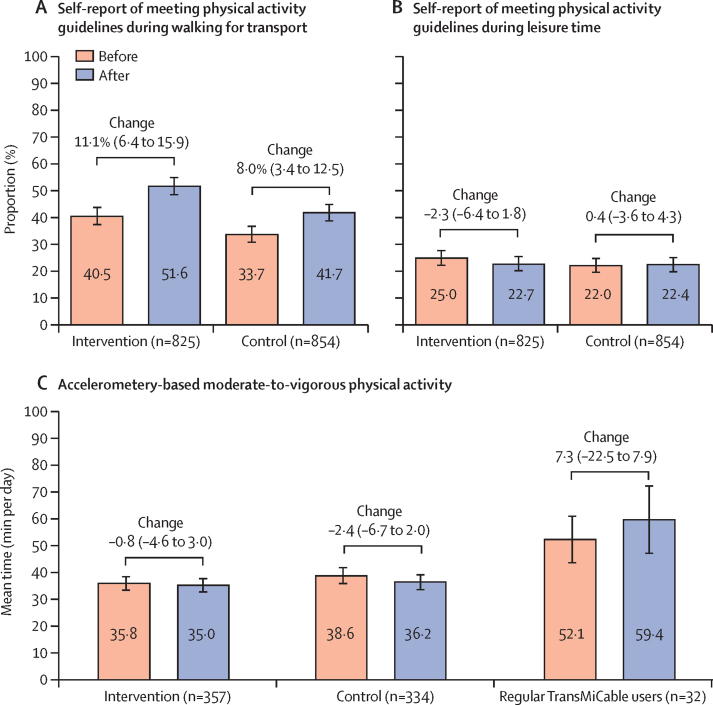
Physical activity levels in the intervention and control areas before and after inauguration of the TransMiCable cable car intervention Error bars are 95% CIs. Change is percentage point difference (95% CI) based on χ^2^ test (parts A and B) or mean difference (95% CI) based on paired *t*-test (part C). Regular TransMiCable users corresponded to 32 (12%) of 357 participants in the intervention group with accelerometery data who reported using TransMiCable on at least one part of mandatory trips (travel for work or education).

The proportions of participants who reported levels of physical activity that met the 2020 WHO guidelines (≥150 min per week of moderate activity, ≥75 min per week of vigorous activity, or equivalent combinations^18^) during transport-related walking are shown in figure 3A. In the intervention group, the proportion of participants who met the guideline-recommended amounts of activity during transport-related walking was 334 (40·5%) of 825 before the TransMiCable inauguration, and 426 (51·6%) after the inauguration (change 11·1 percentage points [95% CI 6·4–15·9]). A similar change was observed in the control group (change 8·0 percentage points [3·4–12·5]; adjusted OR for the time-by-group interaction 1·1 [95% CI 0·8–1·5], p=0·38; appendix 2 p 8).

The proportions of participants who reported levels of physical activity that met the WHO guidelines during leisure time are shown in figure 3B. 206 (25·0%) of 825 participants in the intervention group and 188 (22·0%) of 854 in the control group met the guideline-recommended amounts of activity during leisure time before the TransMiCable inauguration, with no change in the intervention group (187 [22·7%]; change –2·3 percentage points [–6·4 to 1·8]) or control group (191 [22·4%]; change 0·4 percentage points [–3·6 to 4·3]) after the inauguration. Participants in the intervention group did not have an increased likelihood of becoming compliant with the guidelines during leisure time after the TransMiCable inauguration, compared with participants in the control group (adjusted OR for the time-by-group interaction 0·8 [95% CI 0·5–1·2], p=0·26; appendix 2 p 8).

Time spent doing moderate-to-vigorous physical activity as measured with accelerometers is shown in figure 3C. Adults in the intervention group engaged in a mean of 35·8 min per day (SD 26·1) of moderate-to-vigorous physical activity before the TransMiCable inauguration. Time spent doing moderate-to-vigorous physical activity did not change in the intervention group after the inauguration of TransMiCable (35·0 min per day [26·0]; change –0·8 min per day [95% CI –4·6 to 3·0]) and did not change compared with the control group (adjusted β for the time-by-group interaction 1·4 min per day [95% CI –2·0 to 4·9], p=0·41; appendix 2 p 9). The subgroup of regular TransMiCable users (n=32 with accelerometery data) had high moderate-to-vigorous physical activity before the TransMiCable inauguration (52·1 min per day [SD 24·7]) and after the inauguration (59·4 min per day [SD 35·2]; change 7·3 min per day [95% CI –22·5 to 7·9]; figure 3C).

Light physical activity was recorded at a mean of 333·0 min per day (SD 101·4) in the intervention group at baseline, which did not change after the inauguration of TransMiCable (342·1 min per day [107·1]; change 9·1 min per day [95% CI –6·2 to 24·5]) and did not change compared with the control group (adjusted β for the time-by-group interaction 0·7 min per day [95% CI –13·4 to 14·8], p=0·93). Sedentary time in the intervention group was reported at 507·2 min per day (181·1) before the TransMiCable inauguration and 607·9 min per day (230·2) after the inauguration (change 100·7 [70·2 to 131·1]. This change did not differ from the change observed in the control group (adjusted β for the time-by-group interaction –27·6 min per day [–61·7 to 6·5], p=0·11; appendix 2 p 9). Results of the sensitivity analyses with log-transformed outcomes and analyses stratified by sex were overall similar to the main analyses of accelerometery-based activity levels (appendix 2 p 9).

**Figure 4:**
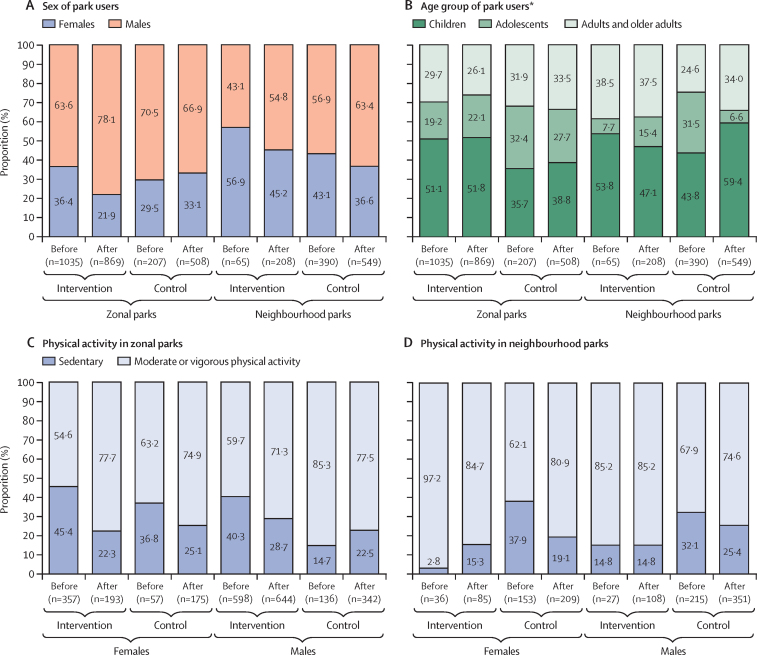
Sex and age characteristics of park users and physical activity levels by sex *Children (age 0–12 years), adolescents (age 13–20 years), adults (age 21–59 years), or older adults (age ≥60 years) according to observer judgement.

In the sample of park users, the sample size before the TransMiCable project was 1035 individuals in the intervention area and 207 individuals in the control area for zonal parks, and 65 individuals in the intervention area and 390 individuals in the control area for neighbourhood parks. After inauguration of the TransMiCable project, the sample size was 869 individuals in the intervention area and 508 individuals in the control area for zonal parks, and 208 individuals in the intervention area and 549 individuals in the control area for neighbourhood parks. We presented the sex and age distributions of the park users (figure 4A–B). In general, the majority of park users in the intervention and control areas were males, and children or adolescents, before and after the TransMiCable inauguration. Figure 4C shows physical activity intensity levels among users of zonal parks. After the TransMiCable intervention, observed moderate or vigorous physical activity in the zonal park of the intervention area increased among females (change 23·1 percentage points [95% CI 15·3 to 30·9]) and males (change 11·6 percentage points [6·3 to 16·8]). In the zonal park of the control area, observed moderate or vigorous physical activity also increased among females (change 11·7 percentage points [–2·4 to 25·8]) but decreased among males (change –7·8 percentage points [–0·4 to 15·2]). After the TransMiCable intervention, male users of the zonal park in the intervention area were more likely to become engaged in moderate or vigorous physical activity than male users of the zonal park in the control area (adjusted OR for the time-by-group interaction 2·7 [95% CI 1·1 to 6·8], p=0·033; appendix 2 p 11). There was no difference among female users (adjusted OR for the time-by-group interaction 0·7 [0·1 to 3·1]; p=0·61).

Physical activity levels among users of neighbourhood parks were also recorded (figure 4D). After the TransMiCable intervention, observed moderate or vigorous physical activity did not change among females (change –12·5 percentage points [95% CI –21·9 to 3·2]) or males (change 0·0 percentage points [–15·0 to 15·0]) in the intervention park. In the control park, observed moderate or vigorous physical activity increased among female users (change 18·8 percentage points [95% CI 9·4 to 28·1]) but did not change among male users (change 6·7 percentage points [–0·9 to 14·5]). After the intervention, female users of the neighbourhood park in the intervention area were less likely to become engaged in moderate or vigorous physical activity than female users of the neighbourhood park in the control area (adjusted OR for the time-by-group interaction 0·4 [95% CI 0·1 to 0·6], p=0·019). There was no difference among male users (adjusted OR for the time-by-group interaction 0·4 [0·1 to 2·6], p=0·36; appendix 2 p 11). appendix 2 (p 10) provides information about the conditions and quality of the parks in the intervention and control areas before and after implementation of the TransMiCable project. The intervention parks had lower quality scores for physical activity provision than the control parks before and after the TransMiCable intervention.

## Discussion

The main objective of this natural experiment was to investigate the effects of the TransMiCable cable car project on physical activity. The main finding was that physical activity remained high with the inauguration of TransMiCable, whether assessed by questionnaire or accelerometery. Indeed, after the inauguration of TransMiCable, 51·6% of adults met guideline-recommended amounts of physical activity just from transport-related walking. Physical activity also increased in one of the renovated parks, but only among male individuals. This study is the first of its kind in Latin America and the comprehensive data allow us to better understand the effects of an integrated urban transport project on physical activity.

Our results at the individual level differ from those reported in studies of high-income areas in the UK (Cambridge) and the USA (Seattle, WA, Salt Lake City, UT, and Los Angeles, CA), and middle-income areas in Mexico (Mexico City), evaluating other transport interventions such as light rail transit and bus rapid transit systems. These interventions have been associated with increased transport-related physical activity and decreased total physical activity.^4^, ^5^ However, physical activity at baseline in these studies (as measured by accelerometery or questionnaire) was reported to be around half of what was found in our study.

Our result that transport-related walking remains high after the implementation of the cable car might be explained by the necessity versus choice-based physical activity framework.^27^ According to this framework, a substantial amount of the physical activity occurring routinely in many low-income settings is the result of economic necessity and is not due to free choices.^27^ In low-income areas, such as the locations in our study, poverty, informal work, crime, and the cost of car ownership make active and multimodal transport a necessity, rather than a choice.^8^, ^13^, ^27^ Studies of low-income settlements in Faridabad (India),^28^ Freetown (Sierra Leone),^29^ and Recife (Brazil)^30^ have indicated that physical activity levels in low-income areas are higher than reported in high-income areas within each country,^28^, ^30^ mainly due to the necessity to walk for transport in low-income areas.^29^ In several Latin American cities, about 30–45% of all trips taken by low-income groups are made on foot, in contrast to around 20% among higher income groups.^8^ Furthermore, when walking as part of other multimodal trips is considered, between 19% and 25% of residents in several Latin American cities have been found to meet physical activity recommendations just from this type of walking.^31^ Therefore, it is encouraging that transport-related walking remained high in our study after the inauguration of the cable car, while travel time for mandatory trips decreased (from 110·0 min to 90·2 min). Furthermore, as reported previously, public transport satisfaction increased (from 4·4 to 5·4 out of 10), and satisfaction for TransMiCable was rated 8·7 out of 10.^11^ These findings are highly relevant especially when an increased number of people in Bogotá were reported to be using private motor vehicles in 2019.^32^

Inhabitants of low-income areas seemingly experience a walkability paradox, as they appear to have high walkability on the basis of prevailing standards (ie, high population density and high street connectivity), but their walkability is poor due to street-level conditions (eg, crime, low-quality or no pavements, unsanitary conditions, pollution, and noise).^27^, ^33^ The 2022 *Lancet Global Health* Series on urban design, transport, and health showed that population density in some low-to-middle-income areas, such as the locations in our study, exceeds optimal thresholds for walking, and there is poor access to public open spaces.^13^, ^14^ In the TrUST study we found that travel time, victimisation, and air pollution in the cabins decreased in the intervention area after the TransMiCable inauguration.^11^, ^34^ However, residents still had long waking hours (from 0400 h to 2400 h), high levels of victimisation, and they are exposed to high air pollution in the bus rapid transit system. Therefore, in low-income populations, where walking for transport is a necessity, efforts to promote physical activity should focus on maintaining regular participation in active travel, while improving the conditions under which it occurs.^27^

We found that children and adolescents were the main users of renovated parks and playgrounds. Drug use in public spaces is a concern,^11^ and we suggest that increased numbers of children and adolescents would use parks and playgrounds if supervision and maintenance were improved. In the present study we also identified an increase in physical activity in one renovated park, but only in boys and men. Lower physical activity levels among girls and women could be related to the infrastructure implemented in parks (eg, football fields and outdoor gyms). Studies in Latin America have shown that community-based physical activity programmes involving dancing increase physical activity among women and should be considered for future interventions in renovated parks.^35^

The results of our study might have important implications for the design and evaluation of urban transport projects that are planned in Bogotá, and in other cities in the Global South. A new public transport system implemented with comprehensive urban interventions has the potential to help towards efforts to maintain high physical activity levels, such as the efforts outlined in the WHO Global Action Plan on Physical Activity 2018–2030.^36^ Such an approach can also help in achieving the UN Sustainable Development Goals.^2^

Our study has several strengths. To the best of our knowledge, TrUST is the only evaluation of the effects of a cable car on physical activity. Also, this study used various methods to assess physical activity at the individual and contextual level. Our study also has limitations that should be considered. First, the accelerometers did not capture the effect of the high terrain slope on physical activity intensity. Second, the final sample sizes were smaller than the target number of 426 adults per group in the accelerometery sample. Third, renovations in the intervention and control parks were outside the control of researchers, and we were unable to collect data in parks early in the morning or late in the day (before 0900 or after 1600) due to concerns about the safety of the investigators.

In conclusion, it is encouraging that physical activity remained high in the intervention area when the use of private motorised transport had increased elsewhere in Bogotá. In low-income populations, where walking is a necessity, urban transport interventions should be accompanied by comprehensive efforts to maintain participation in active travel, while improving the conditions under which this travel takes place.

## Data sharing

The Urban Health in Latin America (SALURBAL) project welcomes queries from anyone interested in learning more about its dataset and potential access to data. Further information about the SALURBAL dataset is available on the SALURBAL project website (www.lacurbanhealth.org) or via email (salurbal@drexel.edu). After publication of this study, the study protocols, data dictionaries, and requested study data may be made available to interested investigators after they have submitted a proposal by email and signed a data use agreement with SALURBAL, and if their study proposal, developed in collaboration with SALURBAL investigators, is approved by the SALURBAL proposal and publications committee. Some data might not be available to external investigators because of data confidentiality agreements.

## Declaration of interests

We declare no competing interests.

## Supplementary Materials

Spanish translation of the abstract

## References

[1] WHO (2020). Physical activity. https://www.who.int/news-room/fact-sheets/detail/physical-activity.

[2] Salvo D, Garcia L, Reis RS (2021). Physical activity promotion and the United Nations Sustainable Development Goals: building synergies to maximize impact. J Phys Act Health.

[3] Guthold R, Stevens GA, Riley LM, Bull FC (2018). Worldwide trends in insufficient physical activity from 2001 to 2016: a pooled analysis of 358 population-based surveys with 1·9 million participants. Lancet Glob Health.

[4] Xiao C, Goryakin Y, Cecchini M (2019). Physical activity levels and new public transit: a systematic review and meta-analysis. Am J Prev Med.

[5] Hirsch JA, DeVries DN, Brauer M, Frank LD, Winters M (2018). Impact of new rapid transit on physical activity: a meta-analysis. Prev Med Rep.

[6] OECD (May 21, 2015). In it together: why less inequality benefits all. https://www.oecd-ilibrary.org/employment/in-it-together-why-less-inequality-benefits-all_9789264235120-en.

[7] Henson RM, Ortigoza A, Martinez-Folgar K (2020). Evaluating the health effects of place-based slum upgrading physical environment interventions: a systematic review (2012–2018). Soc Sci Med.

[8] Rivas ME, Serebrisky T (April, 2021). The role of active transport modes in enhancing the mobility of low-income people in Latin America and the Caribbean. https://publications.iadb.org/en/role-active-transport-modes-enhancing-mobility-low-income-people-latin-america-and-caribbean.

[9] Cano Rubiano L, Gonzalez Portabales I, Lincoln F, Duarte D, Valdiviesco Sierra L (Sept 30, 2020). Urban aerial cable cars as mass transit systems case studies, technical specifications, and business models. https://documents.worldbank.org/en/publication/documents-reports/documentdetail/140251611326011996/urban-aerial-cable-cars-as-mass-transit-systems-case-studies-technical-specifications-and-business-models.

[10] Alcaldía Mayor de Bogotá DC, Transmilenio SA (2017). TansMiCable: un sueño de volar hecho realidad. https://www.caf.com/media/8389/7-transmicable-un-sueno-de-volar-hecho-realidad-jerzon-carrillo.pdf.

[11] Guevara-Aladino P, Baldovino-Chiquillo L, Rubio MA (2023). Winds of change: the case of TransMiCable, a community-engaged transport intervention improving equity and health in Bogotá, Colombia. Cities Health.

[12] Sarmiento OL, Higuera-Mendieta D, Wilches-Mogollon MA (2020). Urban transformations and health: methods for TrUST—a natural experiment evaluating the impacts of a mass transit cable car in Bogotá, Colombia. Front Public Health.

[13] Lowe M, Adlakha D, Sallis JF (2022). City planning policies to support health and sustainability: an international comparison of policy indicators for 25 cities. Lancet Glob Health.

[14] Giles-Corti B, Moudon AV, Lowe M (2022). What next? Expanding our view of city planning and global health, and implementing and monitoring evidence-informed policy. Lancet Glob Health.

[15] Alcaldía Mayor de Bogotá (2019). Decreto 551 de 2019. https://www.alcaldiabogota.gov.co/sisjur/normas/Norma1.jsp?i=86548#.

[16] Secretaría Distrital de Seguridad, Convivencia y Justicia (2019). Boletín mensual de indicadores de seguridad y convivencia por localidad. https://scj.gov.co/es/oficina-oaiee/boletines.

[17] Craig CL, Marshall AL, Sjöström M (2003). International physical activity questionnaire: 12-country reliability and validity. Med Sci Sports Exerc.

[18] WHO (Nov 25, 2020). WHO guidelines on physical activity and sedentary behaviour. https://www.who.int/publications/i/item/9789240015128.

[19] Freedson PS, Melanson E, Sirard J (1998). Calibration of the Computer Science and Applications, Inc. accelerometer. Med Sci Sports Exerc.

[20] McKenzie TL, Cohen DA, Sehgal A, Williamson S, Golinelli D (2006). System for Observing Play and Recreation in Communities (SOPARC): reliability and feasibility measures. J Phys Act Health.

[21] Santos MPM, Rech CR, Alberico CO (2016). Utility and reliability of an app for the System for Observing Play and Recreation in Communities (iSOPARC®). Meas Phys Educ Exerc Sci.

[22] Lee RE, Booth KM, Reese-Smith JY, Regan G, Howard HH (2005). The Physical Activity Resource Assessment (PARA) instrument: evaluating features, amenities and incivilities of physical activity resources in urban neighborhoods. Int J Behav Nutr Phys Act.

[23] Heath GW, Parra DC, Sarmiento OL (2012). Evidence-based intervention in physical activity: lessons from around the world. Lancet.

[24] Ogilvie D, Griffin S, Jones A (2010). Commuting and health in Cambridge: a study of a ‘natural experiment’ in the provision of new transport infrastructure. BMC Public Health.

[25] Hillsdon M, Foster C, Thorogood M (2005). Interventions for promoting physical activity. Cochrane Database Syst Rev.

[26] Ministerio del Trabajo de Colombia Decreto 2269 de 2017. https://www.funcionpublica.gov.co/eva/gestornormativo/norma.php?i=84939.

[27] Salvo D, Jáuregui A, Adlakha D, Sarmiento OL, Reis RS (2023). When moving is the only option: the role of necessity versus choice for understanding and promoting physical activity in low- and middle-income countries. Annu Rev Public Health.

[28] Anand K, Shah B, Yadav K (2007). Are the urban poor vulnerable to non-communicable diseases? A survey of risk factors for non-communicable diseases in urban slums of Faridabad. Natl Med J India.

[29] Oviedo D, Okyere SA, Nieto M (2021). Walking off the beaten path: everyday walking environment and practices in informal settlements in Freetown. Res Transp Bus Manag.

[30] Alves JGB, Figueiroa JN, Alves LV (2011). Prevalence and predictors of physical inactivity in a slum in Brazil. J Urban Health.

[31] Delclòs-Alió X, Rodríguez DA, Medina C (2022). Walking for transportation in large Latin American cities: walking-only trips and total walking events and their sociodemographic correlates. Transp Rev.

[32] Secretaría Distrital de Movilidad (2019). Sistema Integrado de Información sobre Movilidad Urbano Regional: Registro Distrital Automotor. https://www.simur.gov.co/indicadores/transporte-privado.

[33] Guzman LA, Arellana J, Castro WF (2022). Desirable streets for pedestrians: using a street-level index to assess walkability. Transp Res Part D Transp Environ.

[34] Morales-Betancourt R, Wilches-Mogollon MA, Sarmiento OL (2023). Commuter's personal exposure to air pollutants after the implementation of a cable car for public transport: results of the natural experiment TrUST. Sci Total Environ.

[35] Rubio MA, Guevara-Aladino P, Urbano M (2022). Innovative participatory evaluation methodologies to assess and sustain multilevel impacts of two community-based physical activity programs for women in Colombia. BMC Public Health.

[36] WHO (2018).

