# Supplementary material for: Effects of an urban cable car intervention on physical activity: the TrUST natural experiment in Bogotá, Colombia
Source: Lancet Glob Health. 2023 Jul 18;11(8):e1290–300. doi: 10.1016/S2214-109X(23)00274-7 (PMC10369015; doi:10.1016/S2214-109X(23)00274-7)
Supplement: Spanish translation of the abstract [file mmc1.pdf]

THE LANCET
Global Health

Supplementary appendix 1

This translation in Spanish was submitted by the authors and we reproduce it as supplied. It has not been peer reviewed. *The Lancet*'s editorial processes have only been applied to the original in English, which should serve as reference for this manuscript.

Los autores nos proporcionaron esta traducción al español y la reproducimos tal como nos fue entregada. No la hemos revisado. Los procesos editoriales de *The Lancet* se han aplicado únicamente al original en inglés, que debe servir de referencia para este manuscrito.

Supplement to: Baldovino-Chiquillo L, L Sarmiento OL, O'Donovan G, et al. Effects of an urban cable car intervention on physical activity: the TrUST natural experiment in Bogotá, Colombia. *Lancet Glob Health* 2023; **11:** e1290–300.

Efectos de la intervención de un cable aéreo urbano en la actividad física: el experimento natural TrUST en Bogotá, Colombia

Resumen

Introducción: Los cables aéreos hacen parte de los sistemas de transporte en varias ciudades en América Latina. Sin embargo, no hay evaluaciones disponibles sobre sus efectos en la actividad física. TransMiCable es el primer cable aéreo en Bogotá, Colombia, y la intervención urbana más amplia incluye parques renovados. Nosotros evaluamos los efectos de TransMiCable, con su amplia intervención, en la actividad física.

Métodos: El experimento natural Transformaciones Urbanas y Salud (TrUST) fue un estudio prospectivo cuasi experimental realizado desde el 1 de febrero de 2018 hasta el 18 de diciembre de 2018 (línea de base, antes de la intervención) y desde el 2 de julio de 2019 hasta el 15 de marzo del 2020 (seguimiento después de la intervención) en el área de intervención de TransMiCable (localidad de Ciudad Bolívar) y un área de control sin TransMiCable (localidad de San Cristóbal). Se utilizó una estrategia multietápica para muestrear hogares en cada área y se invitó a un adulto (≥18 años) por hogar a participar. Los participantes elegibles habían vivido en el área de intervención o control durante al menos dos años y no tenían planeado mudarse dentro de los siguientes dos años. Se evaluó la actividad física entre los participantes en las áreas de intervención y control antes y después de la inauguración de TransMiCable en Ciudad Bolívar con el Cuestionario Internacional de Actividad Física (formato largo) y con acelerómetros. Los casos completos (aquellos participantes con datos de línea de base y de seguimiento) fueron incluidos en los análisis. Los respondientes fueron clasificados como físicamente activos si cumplían las recomendaciones de la OMS 2020 (≥150 minutos por semana de actividad moderada, ≥75 minutos por semana de actividad vigorosa, o combinaciones equivalentes); y los datos de acelerometría fueron clasificados con los puntajes de corte de Freedson para adultos. Además, se recolectaron datos en parques zonales (área ≥10 000 m2) y parques vecinales (área ≤10 000 m2) en las áreas de intervención y control por observación directa usando el Sistema de Observación del Juego y la Recreación en las Comunidades, para evaluar los niveles de actividad física antes y después de la intervención de TransMiCable. Se usaron modelos de regresión multinivel para evaluar los cambios en la actividad física asociados con la intervención de TransMiCable.

Resultados: El cuestionario de actividad física fue completado por 2052 participantes adultos (1289 [62·8%] mujeres y 763 [37·2%] hombres; edad media 43·5 años [DE 17·7]) antes de la inauguración de TransMiCable. Después de la inauguración, la muestra de seguimiento (final) del cuestionario estaba conformada por 825 adultos en el grupo de intervención y 854 en el grupo de control, incluyendo 357 adultos en el grupo de intervención y 334 en el grupo de control con datos válidos de acelerometría. 334 (40·5%) de los 825 participantes en el grupo de intervención reportaron niveles de actividad física durante la caminata de transporte que cumplían con las recomendaciones de la OMS 2020 antes de la intervención y 426 (51·6%) después de la intervención (cambio 11·1 puntos porcentuales [IC del 95% 6·4 a 15·9]). Un cambio similar se observó en el grupo de control (cambio 8.0 puntos porcentuales [IC 95% 3·4 a 12·5]; OR ajustado para la interacción de tiempo por grupo, grupo de intervención versus grupo de control: 1·1 [IC del 95% 0·8 a 1·5], p=0·38). El tiempo de actividad física moderada a vigorosa, medido con acelerómetros, no cambió en el grupo de

intervención después de la inauguración de TransMiCable (cambio -0.8 minutos por día [-4·6 a 3·0]) y no cambió comparado con el grupo de control (β ajustado para la interacción tiempo por grupo: 1·4 minutos por día [IC del 95% -2·0 a 4·9], p=0·41). La actividad física moderada a vigorosa fue 52·1 minutos por día (DE 24·7) antes y 59·4 minutos por día (35·2) después de la inauguración de TransMiCable en los nuevos usuarios regulares que reportaron usar TransMiCable para viajes obligados para ir al trabajo o lugar de estudio (n=32; cambio 7·3 minutos por día [-22·5, 7·9]). Después de la intervención, se observó un incremento en la proporción de usuarios hombres que realizaban actividad física moderada o vigorosa en el parque zonal renovado (OR ajustado para la interacción tiempo por grupo, parque de intervención versus parque de control: 2·7 [1·1 a 6·8], p=0.033). También se observó que las usuarias mujeres del parque vecinal renovado tuvieron menos posibilidades de realizar actividad física moderada a vigorosa que las usuarias mujeres del parque vecinal del área de control (OR ajustado para la interacción tiempo por grupo: 0·4 [0·1 a 0·6], p=0·019).

Interpretación: Es esperanzador que la caminata relacionada con el transporte se mantuviera alta en el área de intervención de TransMiCable cuando el uso de transporte privado motorizado ha aumentado en Bogotá. En las áreas urbanas de bajos ingresos, donde los desplazamientos a pie son una necesidad, las intervenciones en transporte deben enfocarse en esfuerzos para mantener la participación en viajes activos, al mismo tiempo que se mejoren las condiciones en las cuales estos viajes ocurren.

Financiamiento: Wellcome Trust (como parte del proyecto Salud Urbana en América Latina); Secretaría Distrital de Planeación de Bogotá; Ministerio de Ciencias, Tecnología e Innovación de Colombia; Universidad de Los Andes; Fundación Santa Fe de Bogotá; y Universidad del Norte.
