# Supplementary material for: Effects of an urban cable car intervention on physical activity: the TrUST natural experiment in Bogotá, Colombia
Source: Lancet Glob Health. 2023 Jul 18;11(8):e1290–300. doi: 10.1016/S2214-109X(23)00274-7 (PMC10369015; doi:10.1016/S2214-109X(23)00274-7)
Supplement: Supplementary appendix 2 [file mmc2.pdf]

THE LANCET
Global Health

Supplementary appendix 2

This appendix formed part of the original submission and has been peer reviewed. We post it as supplied by the authors.

Supplement to: Baldovino-Chiquillo L, L Sarmiento OL, O'Donovan G, et al. Effects of an urban cable car intervention on physical activity: the TrUST natural experiment in Bogotá, Colombia. *Lancet Glob Health* 2023; **11:** e1290–300.

Supplementary appendix

Effects of an urban cable car intervention on physical activity: the TrUST natural experiment in Bogotá, Colombia

Laura Baldovino-Chiquillo MSc[1], Olga L. Sarmiento PhD[1], Gary O'Donovan PhD[1,2,3], Maria A. Wilches-Mogollon PhD[1,4], Andres F. Aguilar BSc[1], Alberto Florez-Pregonero PhD[5], Paola A. Martínez BSc[1], Julian Arellana PhD[6], Luis A. Guzmán PhD[7], Goro Yamada PhD[8], Daniel A. Rodriguez PhD[9], Ana V. Diez-Roux PhD[8,10].

[1] School of Medicine, Universidad de los Andes, Bogotá, Colombia
[2] BrainLat, Universidad Adolfo Ibáñez, Santiago, Chile
[3] Instituto Masira, Universidad de Santander, Bucaramanga, Colombia
[4] Department of Industrial Engineering, School of Engineering, Universidad de los Andes, Bogotá, Colombia
[5] School of Education, Pontificia Universidad Javeriana, Bogotá, Colombia
[6] Department of Civil and Environmental Engineering, Universidad del Norte, Barranquilla, Colombia
[7] Department of Civil and Environmental Engineering, School of Engineering, Universidad de los Andes, Bogotá, Colombia
[8] Urban Health Collaborative, Dornsife School of Public Health, Drexel University, Philadelphia, PA, USA
[9] Department of City and Regional Planning, and Institute for Transport Studies, University of California Berkeley, Berkeley, CA, USA
[10] Department of Epidemiology and Biostatistics, Dornsife School of Public Health, Drexel University, Philadelphia, PA, USA

Corresponding author: Olga L. Sarmiento, osarmien@uniandes.edu.co, Universidad de Los Andes, Carrera 1 N° 18A-12, Bogotá – Colombia, Phone: (57-1) 3394949 ext 3785

Table of contents

Study setting and the TransMiCable intervention 3

Figure S1. Map of the location of the intervention and control areas in Bogotá. 3

Figure S2. The TransMiCable intervention. The TrUST study, 2018-2020. 4

Figure S3. Sampling buffer areas and evaluated parks. The TrUST study, 2018-2020. 5

Figure S4. Timeline of measurements in adults and parks of the TrUST study, 2018-2020. 5

Training of observers for the application of the System for Observing Play and Recreation in Communities 6

Methods for the quality score of the park's infrastructure 6

Equation form for the multilevel regression models at the individual level 6

Equation form for the multilevel regression models at the park level 7

Table S1. The effects of the implementation of TransMiCable on meeting physical activity recommendations during walking for transport and leisure time. The TrUST study, 2018-2020 8

Table S2. The effects of the implementation of TransMiCable on minutes per day of moderate-to-vigorous physical activity among individuals. The TrUST study 2018-2020. 9

Table S3. Characteristics and quality of parks. The TrUST study, 2018-2020. 10

Table S4. The effects of the park's renovation on occupancy and physical activity in the parks. The TrUST study, 2018-2020. 11

Study setting and the TransMiCable intervention

In the figure S1, the map shows the location, distance and geographical barriers of the intervention and control areas in the south of Bogotá. Additionally, it shows the different transport routes to the bus rapid transit system for the intervention and control areas.

Additional photos of the TransMiCable intervention are shown in figure S2. Furthermore, a video about the TransMiCable intervention from the perspective of two community leaders from Ciudad Bolivar is available at this link of the *Salud Urbana en América Latina* (SALURBAL) project: https://www.youtube.com/watch?v=4dvQ2NPikU4

Figure S1. Map of the location of the intervention and control areas in Bogotá.
The stations outside the intervention and control areas correspond to connection stations (already existed before the cable car) with the Bus Rapid Transit System of Bogotá (TransMilenio, 2002). This map was created in ArcGIS (version 10.3).

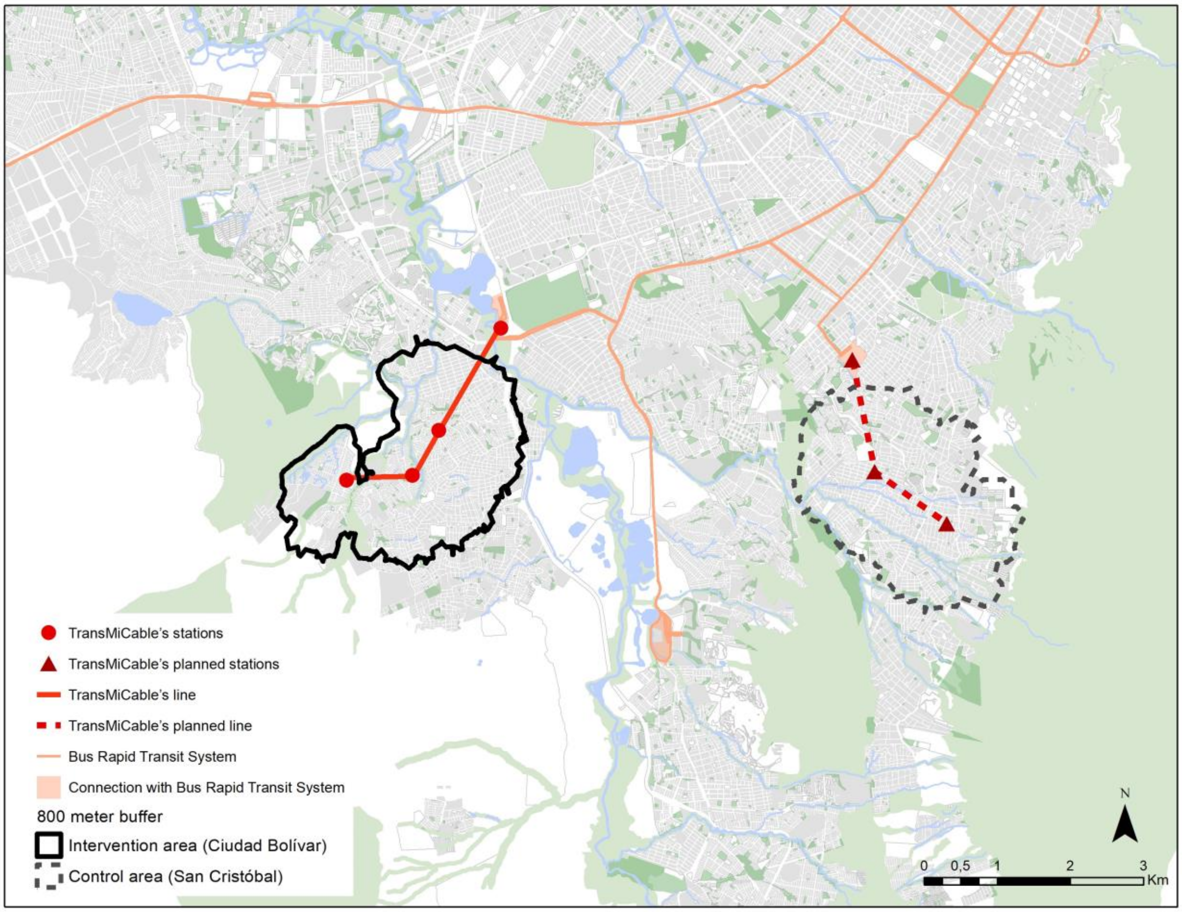

Figure S2. The TransMiCable intervention. The TrUST study, 2018-2020.
The TransMiCable cable car system in Ciudad Bolívar (A) and the parks and playgrounds that were implemented in the Illimaní park as part of the wider intervention (B).

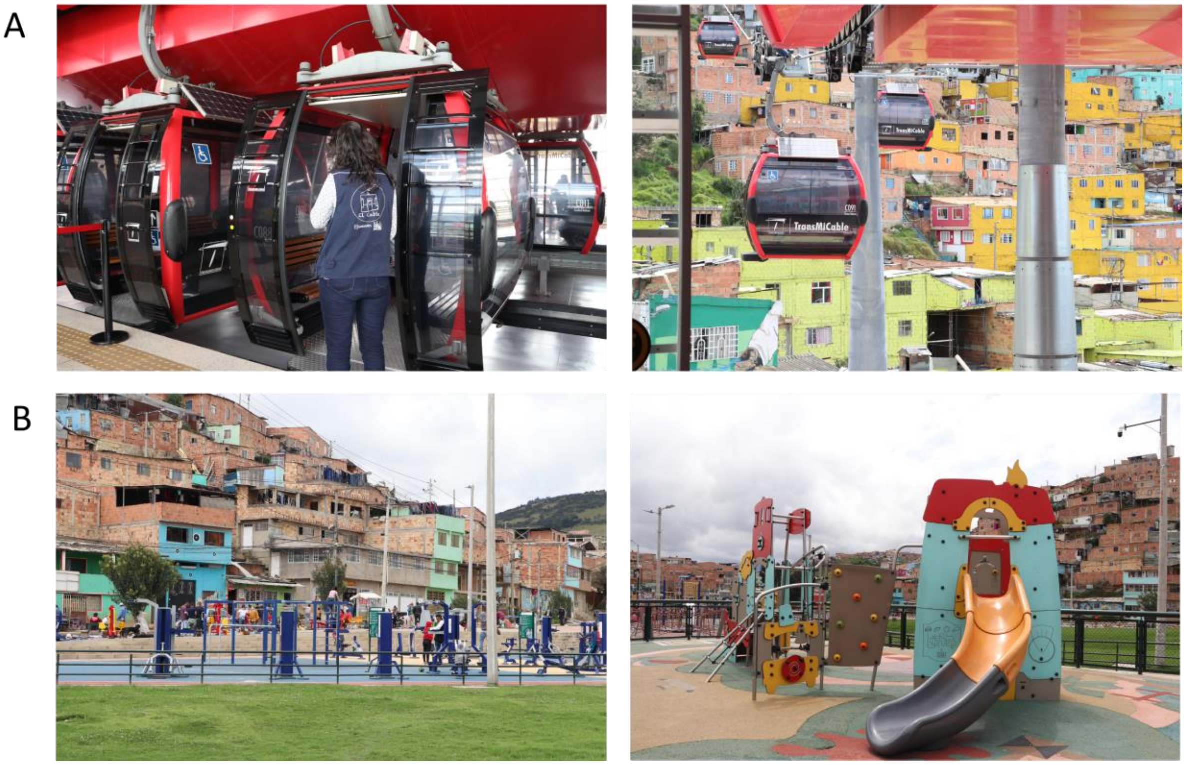

Figure S3. Sampling buffer areas and evaluated parks. The TrUST study, 2018-2020.
Maps of the sampling buffer areas and evaluated parks in the intervention (A, left), and control (B, right) groups. The stations outside the intervention and control areas correspond to connection stations (already existed before the cable car) with the Bus Rapid Transit System of Bogotá (TransMilenio, 2002). This map was created in ArcGIS (version 10.3).

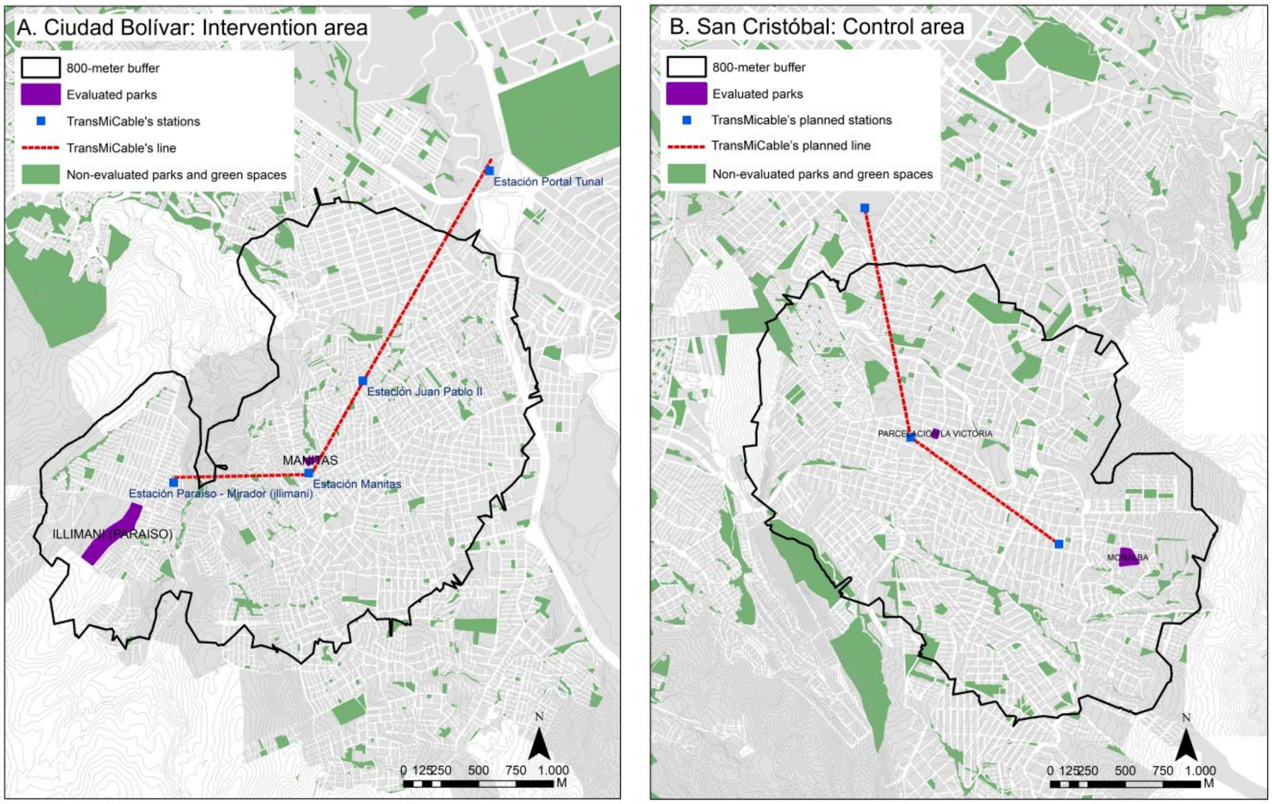

Figure S4. Timeline of measurements in adults and parks of the TrUST study, 2018-2020.

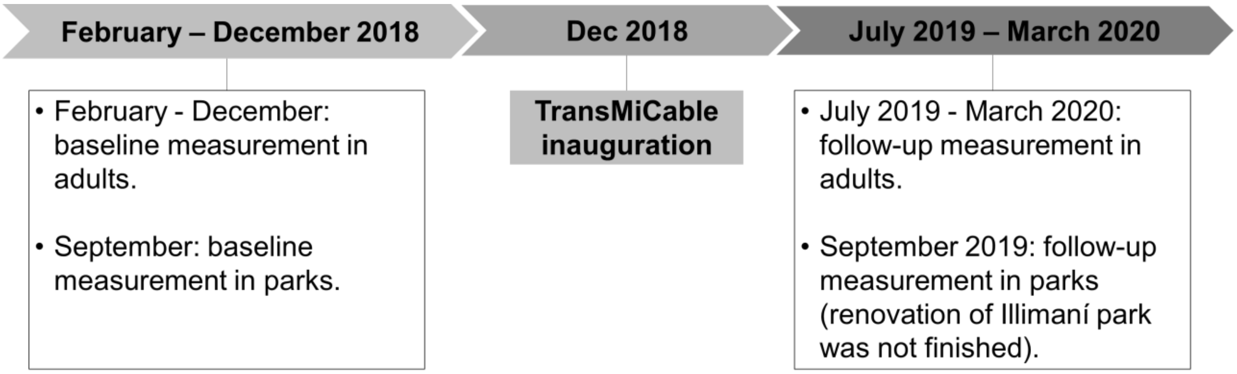

Training of observers for the application of the System for Observing Play and Recreation in Communities

Two independent observers conducted the measurements in the four evaluated parks before and after the TransMiCable project. The observers were trained in each measurement by researchers who are experts in the use of the System for Observing Play and Recreation in Communities (SOPARC), including theoretical and practical components. The theoretical component was conducted during a one-day workshop (three hours) during each measurement. During these workshops, observers were familiarized with the SOPARC method (operational definitions, instrument, coding, and categorization of the physical activity levels and age groups by gender). In addition, instructions for registering and completing the SOPARC form using a mechanical counter were also given. SOPARC training materials, available on the Active Living Research website (https://activelivingresearch.org/), were used for training the observers. The practical component was conducted over two days (12 hours) before the implementation of TransMiCable and one day (10 hours) afterwards in the four parks, comparing results between observers and receiving feedback from the coordinators.

We also evaluated the concordance of the measures between the two observers in both the baseline and follow-up measurements. Specifically, the intraclass coefficient was calculated to estimate the inter-observer reliability on observations of users according to sex, age group, and physical activity level. In general, we estimated the intraclass coefficient ranged between 0·884 and 0·998 in the baseline measurements and between 0·970 and 0·998 in the follow-up measurement.

Methods for the quality score of the park's infrastructure

The quality of the park's infrastructure was assessed with the Physical Activity Resource Assessment (PARA) instrument. PARA qualifies the conditions of parks into six domains. 1) Features for physical activity, including the following items: fields and court sports, exercise area, trails for walking, running, skating, roller-skating, swimming pools, and playgrounds; 2) Amenities, including bathrooms, benches, locker rooms, lighting, trash cans, and picnic tables; 3) Incivilities, including cleanliness, aesthetics, safety, dog refuse, garbage, broken glass, graffiti/tagging, vandalism, overgrown grass, unrestrained dogs, litter; 4) Services, including restaurants, libraries, physical activity materials, physical activity classes; 5) Accessibility, including taxi and bus stops, parking, bike racks, and bicycle paths; and 6) Safety. The items of features and amenities domains were classified into four categories: "not present" (points: 0), "poor" (points: 1), "mediocre" (points: 2), and "good" (points: 3). The items of incivilities domain were classified as "not present" (points: 3), "small presence" (points: 2), "average presence" (points: 1), or "very present" (points: 0). The items of services, accessibility, and safety domains were classified as "absence" (points: 0) and "presence" (points: 1). The total score of parks' quality was computed as follow: points for features + amenities - incivilities + accessibility + safety.

Equation form for the multilevel regression models at the individual level

The form of the multilevel linear regression models at the individual level is shown in Equation (1) and the formof the multilevel logistic regression models is shown in Equation (2).

$$Y_{it} = \beta_0 + \beta_1 Time_{it} + \beta_2 Treatment_i + \beta_3 (Time_{it} * Treatment_i) + \theta X_{it} + \xi Z_i + u_i + e_{it} \quad (1)$$

$$logit(P(Y_{it} = 1)) = \beta_0 + \beta_1 Time_{it} + \beta_2 Treatment_i + \beta_3 (Time_{it} * Treatment_i) + \theta X_{it} + \xi Z_i + u_i \quad (2)$$

Where:

Y_{it} is the physical activity outcomes for individual i that was observed during two periods t (t = T_0, T_1).
β_0 is the overall intercept (average across individuals).
$Time_{it}$ is the period at which measurement t was taken on individual i. T_0 denotes the pre-intervention period and T_1 is the post-intervention period.
$Treatment_i$ is the treatment condition dummy variable, equal to 1 for individuals living in Ciudad Bolivar (intervention group) and 0 for individuals living in San Cristóbal (control group).
$Time_{it} * Treatment_i$ is the interaction term used to assess the effect of TransMiCable by comparing the

changes in outcomes over time between the intervention and control groups.
X_{it} are individual characteristics that vary on time (age, sex, occupation, education, marital status, and distance to the Bus Rapid Transit system).
Z_i is a neighborhood characteristic (terrain slope) that varies among individuals.
$u_i \sim N(0, \sigma_u^2)$ is an individual-specific random effect (between-individual variance in y).
$e_{it} \sim N(0, \sigma_e^2)$ is a time-varying residual (within-individual variance y).

Equation form for the multilevel regression models at the park level

The form of the multilevel regression models at the park level is shown in Equation (3).

$$logit(P(Y_{pt} = 1)) = \beta_0 + \beta_1 Time_{pt} + \beta_2 Treatment_p + \beta_3 (Time_{pt} * Treatment_p) + \theta X_{pt} + u_p \quad (3)$$

Where:

Y_{pt} is the physical activity outcome for a park p that was observed during two periods t (t = T_0, T_1).
β_0 is the overall intercept (average across parks).
$Time_{pt}$ is the period at which measurement t was taken on park p. T_0 denotes the pre-intervention period and T_1 is the post-intervention period.
$Treatment_p$ is the treatment condition dummy variable, equal to 1 the park in Ciudad Bolivar (intervention area) and 0 for the park in San Cristóbal (control area).
$Time_{pt} * Treatment_p$ is the interaction term used to assess the effect of the park's renovation implemented in the context of the TransMiCable project by comparing the changes in outcomes over time between the intervention and control groups.
X_p is the day of the week (weekdays or weekend days) in which was observed the park.
$u_p \sim N(0, \sigma_u^2)$ is a park-specific random effect according to the target area, day of observation, and period of the day in which was observed each park (between-park variance in y).

Table S1. Effects of the implementation of TransMiCable on meeting physical activity recommendations during walking for transport and leisure time. The TrUST study, 2018-2020.

	**Intervention (N=825)[a]**			**Control (N=854)[a]**			**Unadjusted multilevel logistic regression model (Time by group interaction)**			**Adjusted multilevel logistic regression model[b] (Time by group interaction)**		
	**Before**	**After**	**Change**	**Before**	**After**	**Change**						
	**N (%)**	**N (%)**		**N (%)**	**N (%)**		**Odds Ratio**	**95% CI**	***p***	**Odds Ratio**	**95% CI**	***p***
Overall												
Walking for transport	334 (40·5)	426 (51·6)	+11·1	288 (33·7)	356 (41·7)	+8·0	1·14	(0·85;1·55)	0·38	1·15	(0·85;1·55)	0·38
Leisure time	206 (25·0)	187 (22·7)	-2·3	188 (22·0)	191 (22·4)	+0·4	0·80	(0·54;1·19)	0·27	0·79	(0·54;1·18)	0·26
Females												
Walking for transport	216 (40·0)	289 (53·5)	+13·5	160 (30·5)	222 (42·4)	+11·8	1·05	(0·71;1·55)	0·81	1·05	(0·71;1·55)	0·81
Leisure time	103 (19·1)	97 (18·0)	-1·1	84 (16·0)	88 (16·8)	+0·8	0·83	(0·50;1·42)	0·50	0·82	(0·48;1·39)	0·46
Males												
Walking for transport	118 (41·4)	137 (48·1)	+6·7	128 (38·8)	134 (40·6)	+1·8	1·24	(0·77;2·00)	0·37	1·24	(0·77;2·01)	0·37
Leisure time	103 (36·1)	90 (31·6)	-4·5	104 (31·5)	103 (31·2)	-0·3	0·75	(0·42;1·35)	0·35	0·76	(0·42;1·36)	0·35

a. Complete case sample including only participants who answered the follow-up survey.
b. Multilevel logistic regression model with random intercepts for individuals, adjusted by age, sex, occupation, marital status, education, distance to bus rapid transit, and the slope of the terrain.

Table S2. Effects of the implementation of TransMiCable on minutes per day of moderate-to-vigorous physical activity among individuals. The TrUST study 2018-2020.

	Intervention (N=357)[a]			Control (N=334)[a]			Unadjusted multilevel linear regression model (Time by group interaction)			Adjusted multilevel linear regression model[b] (Time by group interaction)			Ln multilevel linear regression model[c] (Time by group interaction)		
	Before	After	Change	Before	After	Change									
	Mean (SD)	Mean (SD)		Mean (SD)	Mean (SD)		β	95% CI	*p*	β	95% CI	*p*	B_{ln}	95% CI	*p*
Overall	N=357			N=334											
MVPA	35·8 (26·1)	35·0 (26·0)	-0·8	38·6 (29·5)	36·2 (27·6)	-2·4	1·53	(-1·92;4·97)	0·39	1·45	(-2·02;4·93)	0·41	0·04	(-0·09;0·16)	0·56
LPA	333·0 (101·4)	342·1 (107·1)	+9·1	329·9 (100·1)	339·1 (99·9)	+9·2	-0·04	(-14·04;13·96)	0·99	0·67	(-13·43;14·77)	0·93	-0·02	(-0·07;0·03)	0·36
SED	507·2 (181·1)	607·9 (230·2)	+100·7	511·2 (191·3)	638·1 (232·2)	+126·9	-26·30	(-60·30;7·71)	0·13	-27·61	(-61·74;6·53)	0·11	-0·05	(-0·11;0·01)	0·08
Females	N=228			N=200											
MVPA	31·8 (23·4)	31·6 (24·5)	-0·2	33·7 (24·2)	31·6 (22·1)	-2·1	1·90	(-1·82;5·64)	0·32	1·82	(-1·94;5·59)	0·34	0·03	(-0·12;0·19)	0·68
LPA	341·2 (97·1)	352·0 (104·1)	+10·8	346·5 (96·7)	349·7 (101·7)	+3·2	7·71	(-9·56;24·97)	0·38	8·59	(-8·81;25·99)	0·33	0·02	(-0·04;0·07)	0·60
SED	497·5 (177·2)	587·1 (222·5)	+89·6	497·1 (193·4)	627·7 (232·4)	+130·6	-40·96	(-83·41;1·49)	0·06	-40·65	(-83·53;2·23)	0·06	-0·08	(-0·15;-0·01)	0·03
Males	N=129			N=134											
MVPA	42·9 (29·0)	41·0 (27·6)	-1·9	45·9 (34·9)	43·2 (33·1)	-2·7	0·80	(-5·90;7·49)	0·82	0·74	(-6·04;7·53)	0·83	0·04	(-0·16;0·24)	0·70
LPA	318·6 (107·6)	324·6 (110·3)	+6·0	305·2 (100·5)	323·3 (95·4)	+18·1	-12·08	(-35·89;11·73)	0·32	-11·68	(-35·79;12·42)	0·34	-0·08	(-0·16;0·01)	0·08
SED	524·4 (187·2)	644·5 (239·6)	+120·1	532·1 (186·8)	653·6 (231·9)	+121·5	-1·38	(-58·02;55·26)	0·96	-5·24	(-62·12;51·64)	0·86	-0·01	(-0·10;0·09)	0·88

Abbreviations: SD standard deviation. MVPA moderate-to-vigorous physical activity. LPA light physical activity. SED sedentary time.

a. Complete case sample including only participants who complete the follow-up with valid accelerometry data.

b. Multilevel linear regression models with random intercepts for individuals, adjusted by age, sex, occupation, marital status, education, distance to bus rapid transit, and the slope of the terrain.

c. Adjusted multilevel linear regression models using log-transformed outcome.

Table S3. Characteristics and quality of parks. The TrUST study, 2018-2020.

	Zonal Parks				Neighbourhood Parks			
	Intervention		Control		Intervention		Control	
	Before	After	Before	After	Before	After	Before	After
**Size of park, m^2**	16000		10000		1600		1100	
**Characteristics of target areas**								
Number	22		15		4		8	
Mean size ± SD, m^2	284·7 ± 419·0		118 ± 72·5		41·5 ± 13·8		66·9 ± 47·8	
Minimum-maximum size, m^2	2·3 - 1127·5		6·8 - 243·9		28·2 - 60·9		9·2 - 110·2	
**Type of area, %**								
Sports area	22·7		40·0		0·0		50·0	
Playground area	54·5		20·0		50·0		25·0	
Open area	13·6		20·0		50·0		25·0	
Walking/running track	4·5		6·6		0·0		0·0	
Strength/stretching exercise area	4·5		13·3		0·0		0·0	
**Conditions of target areas, %**								
Accessible	81·5	13·3	100·0	100·0	100·0	100·0	100·0	100·0
Usable	99·7	31·8	98·1	100·0	100·0	100·0	100·0	100·0
Equipped	52·9	100·0	39·5	37·6	69·6	98·2	25·9	24·1
Supervised	18·2	19·8	1·0	0·5	19·6	57·1	16·1	41·1
Organised activities	9·7	11·4	4·8	1·0	0·0	0·0	11·6	6·3
Empty	37·0	78·6	67·6	61·9	55·4	51·8	24·1	38·4
**Quality score of parks (a)**								
Features for physical activity	8	5	12	13	1	3	5	6
Amenities	9	13	9	9	4	5	8	5
Incivilities (cleaning, aesthetics)	12	11	6	13	8	16	9	16
Services	2	3	1	0	0	0	3	2
Accessibility	3	2	1	2	1	2	3	1
Safety	0	0	0	0	0	0	0	0
Total score (b)	10	12	17	11	-2	-6	10	-2

Abbreviations: SD standard deviation.

a. The quality of the park's infrastructure was assessed with the Physical Activity Resource Assessment (PARA) instrument.

b. Total score = Features + Amenities – Incivilities + Services + Accessibility + Safety.

Table S4. Effects of the park's renovation on occupancy and physical activity in the parks. The TrUST study, 2018-2020.

	Zonal parks[a]									Neighbourhood parks[a]								
	Intervention			Control			Adjusted multilevel logistic regression model[b] (Time by group interaction)			Intervention			Control			Adjusted multilevel logistic regression model[b] (Time by group interaction)		
	Before	After	Change	Before	After	Change	OR	95% CI	*p*	Before	After	Change	Before	After	Change	OR	95% CI	*p*
**Occupancy**																		
Total users, N	1035	869	··	207	508	··	··	··	··	65	208	··	390	549	··	··	··	··
Sex, n (%)																		
Female	377 (36·4)	190 (21·9)	-14·5	61 (29·5)	168 (33·1)	+3·6	1·0	Reference group		37 (56·9)	(94) 45·2	-11·7	168 (43·1)	201 (36·6)	-6·5	1·0	Reference group	
Male	658 (63·6)	679 (78·1)	+14·5	146 (70·5)	340 (66·9)	-3·6	0·6	(0·3;1·1)	0·11	28 (43·1)	114 (54·8)	+11·7	222 (56·9)	348 (63·4)	+6·5	0·9	(0·4;1·8)	0·74
Age group, n (%)																		
Children or adolescent	728 (70·3)	642 (73·9)	+3·6	141 (68·1)	338 (66·5)	+1·5	1·0	Reference group		40 (61·5)	130 (62·5)	+1·0	294 (75·4)	362 (66·0)	-9·3	1·0	Reference group	
Adults or older adults	307 (29·6)	227 (26·1)	-3·5	66 (31·9)	170 (33·5)	+1·6	1·6	(0·4;1·5)	0·51	25 (38·5)	78 (37·5)	-0·9	96 (24·6)	187 (34·0)	+9·5	0·5	(0·2;1·2)	0·13
**Physical activity**																		
Overall																		
Number	955	837	··	193	517	··	··	··	··	63	193	··	368	560	··	··	··	··
Sedentary, %	403 (42·2)	228 (27·2)	-15·0	41 (21·2)	121 (23·4)	-2·2	1·0	Reference group		5 (7·9)	29 (15·0)	+7·1	127 (34·5)	129 (23·0)	-11·5	1·0	Reference group	
MVPA, %	552 (57·8)	609 (72·8)	+15·0	152 (78·8)	396 (76·6)	+2·2	1·6	(0·8;3·3)	0·18	58 (92·1)	164 (85·0)	-7·1	241 (65·5)	431 (77·0)	+11·5	0·2	(0·0;0·7)	0·02
Females																		
Number	357	193	··	57	175	··				36	85		153	209	··	··	··	··
Sedentary, %	162 (45·4)	43 (22·3)	-23·1	21 (36·8)	44 (25·1)	-11·7	1·0	Reference group		1 (2·8)	13 (15·3)	+12·5	58 (37·9)	40 (19·1)	-18·8	1·0	Reference group	
MVPA, %	195 (54·6)	150 (77·7)	+23·1	36 (63·2)	131 (74·9)	+11·7	0·7	(0·1;3·1)	0·61	35 (97·2)	72 (84·7)	-12·5	95 (62·1)	169 (80·9)	+18·8	0·4	(0·1;0·6)	0·02
Males																		
Number	598	644	··	136	342	··	··	··	··	27	108		215	351	··	··	··	··
Sedentary, %	241 (40·3)	185 (28·7)	-11·6	20 (14·7)	77 (22·5)	+7·8	1·0	Reference group		4 (14·8)	16 (14·8)	0·0	69 (32·1)	89 (25·4)	-6·7	1·0	Reference group	
MVPA, %	357 (59·7)	459 (71·3)	+11·6	116 (85·3)	265 (77·5)	-7·8	2·7	(1·1;6·8)	0·03	23 (85·2)	92 (85·2)	0·0	146 (67·9)	262 (74·6)	+6·7	0·4	(0·1;2·6)	0·36

Abbreviations: MVPA moderate or vigorous physical activity. OR Odds Ratio

a. Data come from 14 observations at each target area of the parks (during the morning and the afternoon of seven days of the week).

b. Multilevel logistic regression model with random intercepts for target area, day of observation, and period of the day, adjusted by day of the week (weekdays vs. weekend days).
